# Ginsenosides in the Root Exudates of Ginseng Infected with Rusty Root Rot Improve the Infectivity of Pathogenic *Ilyonectria* Fungi

**DOI:** 10.3390/microorganisms14071484

**Published:** 2026-07-07

**Authors:** Yumeng Song, Wei Li, Xinru Wang, Juan Hua, Shihong Luo

**Affiliations:** College of Bioscience and Biotechnology, Shenyang Agricultural University, Shenyang 110866, China; 15524112155@163.com (Y.S.);

**Keywords:** fungal pathogen, ginsenosides, *Ilyonectria* spp., *Panax ginseng*, rusty root rot

## Abstract

Rusty root rot of ginseng (*Panax ginseng*) caused by *Ilyonectria* spp. infection is a devastating soil-borne disease restricting the sustainable production of garden-cultivated ginseng (GCG) in Northeast China and causes severe yield and economic losses; GCG is far more susceptible to this pathogen than forest-cultivated ginseng (Lin-Xia-Shan-Shen, LXSS). Ginsenosides, the signature triterpenoid saponin defensive metabolites of ginseng, are characteristic dammarane-type triterpenoid defensive saponins represented by Re, Rg_2_, Rb_1_, Rd, and Rg_1_. These compounds are continuously secreted into the rhizosphere and widely participate in plant–microbe interactions, yet their functional roles in mediating *Ilyonectria* infection remain poorly clarified. This study aimed to clarify how rhizospheric ginsenosides regulate the infection process of pathogenic Ilyonectria strains. Two pathogenic strains, *Ilyonectria* sp. SYM-1 and *Ilyonectria* sp. SYM-2, were found isolated from diseased GCG roots and verified as causal agents via morphological observation, molecular ITS identification and artificial inoculation infection experiments. Interestingly, the concentrations of five ginsenosides, Re, Rg_2_, Rb_1_, Rd, and Rg_1_, in the rhizospheric soil of GCG with rusty root rot were significantly higher than those in the rhizospheric soil of healthy LXSS plants. In addition, the concentrations of ginsenosides in the LXSS rhizospheric soils decreased with increasing age of plants. Non-nutritive suspension co-culture assays showed that high concentrations of the ginsenosides Rg_1_ and Rd significantly promoted spore germination of the strains SYM-1 and SYM-2. However, Rb_1_ had a certain inhibitory effect on the growth of *Ilyonectria* sp. SYM-2. Host inoculation experiments further indicated that infection with either fungus significantly reduced the concentrations of ginsenosides produced in ginseng roots. These results demonstrate that the pathogenic fungi SYM-1 and SYM-2 of *Ilyonectria* can adapt to and utilize ginsenosides. Collectively, these findings prove that the two pathogenic *Ilyonectria* strains have evolved the capacity to adapt to and exploit rhizospheric ginsenosides to facilitate their infectivity. From an application perspective, reducing rhizospheric ginsenoside release may represent a promising theoretical strategy for ginseng cultivation and germplasm improvement, which warrants further verification by field or greenhouse experiments for validation.

## 1. Introduction

Ginseng (*Panax ginseng*) is known as the “king of herbal medicine” in China and is important to Chinese traditional medicine [1,2]. Over-collection of wild resources has induced an extreme shortage of pure wild plants, and cultivation has become increasingly important in the ginseng industry. “Lin-Xia-Shan-Shen” (LXSS) is a type of ginseng that is cultivated in the shade in montane forests and is believed to have excellent medicinal properties [3,4]. Indeed, the commodity value of LXSS is higher than that of garden-cultivated ginseng (GCG) in the same year of cultivation [5,6]. However, GCG suffers far more frequent biotic stresses than LXSS; its pathogens include bacteria, fungi and nematodes, seriously restricting cultivation efficiency [7]. Although GCG achieves higher yield, recurrent disease outbreaks have become a bottleneck limiting its sustainable production.

As the core bioactive metabolites of ginseng, ginsenosides mediate critical plant–rhizosphere pathogen crosstalk and bridge the gap between ginseng cultivation and disease occurrence. Ginsenosides are triterpenoid saponins distributed throughout ginseng tissues, mainly categorized into dammarane-type and oleanane-type groups, with Rb_1_, Rb_2_, Rc, Rd, Re and Rg_1_ as major monomers [8,9,10,11,12]. Currently, ginsenosides are shown to be the main chemical substances released by ginseng to the environment [13]. Endogenously, ginsenosides act as defensive compounds to resist pathogen invasion within ginseng tissues. Once released into soil, however, monomers such as Rg1 and Rd act as chemical cues to stimulate spore germination and virulence of *Ilyonectria* pathogens, reflecting their dual regulatory roles: disease resistance inside plants and pathogenicity-promoting signals in the rhizosphere [14].

To further interpret this dual effect, it is necessary to clarify the regulatory relationship between rhizosphere ginsenosides and soil microbial communities. Root exudates rich in ginsenosides shape rhizosphere microbial assembly, and forest cultivation conditions generally support more diverse rhizosphere fungal populations [15,16,17]. Pathogens of soil origin have caused enormous economic losses to China’s agricultural production due to their rapid incidence and the difficulties facing their control. Ginseng is able to inhibit the growth of soil-borne pathogenic fungi through the chemical defense function of its ginsenosides. However, certain pathogenic microorganisms can identify and utilize ginsenosides, causing long-term infections and harming the plants [18]. Therefore, the interactions between ginseng and pathogenic fungi through ginsenosides is a major focus of research into the process of ginseng cultivation.

Ginseng rusty root rot (GRR) is a common fungal disease of ginseng affecting the continuous planting and economic value of ginseng cultivated in gardens and fields [19]. The formation of GRR is a complex process and is influenced by many factors but is mainly due to microorganisms in the soil that infect the plant roots [20]. Rusty root rot infection can lead to 30% reductions in the yield of ginseng grown under garden cultivation, and thus this disease has caused huge economic losses to farmers in China [21]. *Ilyonectria* spp. is the dominant fungus causing rusty root rot and is a species complex containing several important soil-borne pathogens [22]. Ginseng roots infected by *Ilyonectria* spp. fungi will initially display lesions, which then expand to become severe rusty root rot [23]. Tonghua, a major ginseng-producing region in China, has been seriously threatened by ginseng rusty root rot [24]. Although chemical, biological, and cultivation-based strategies have been developed to control GRR, these methods have obvious limitations. Chemical fungicides risk inducing pathogen resistance, environmental pollution, and pesticide residues. Biological control agents often show unstable efficacy in field conditions. Agronomic measures such as crop rotation are restricted by long growth cycles and limited land resources. Thus, effective and sustainable control strategies for GRR are still lacking.

In order to cope with this increasingly serious problem, the purpose of this study was to isolate the pathogenic fungi causing ginseng rusty root rot, to qualitatively and quantitatively analyze ginsenosides in the root exudates of susceptible patients, and to screen their biological activities against pathogenic fungi, so as to reveal the potential biological functions of ginsenosides in the interaction between rusty root rot pathogens and ginseng plants.

## 2. Materials and Methods

### 2.1. Field Investigation and Collection of Ginseng

Field surveys of garden-cultivated ginseng (GCG) were conducted in Jilin Province from July to August 2022 to investigate rusty root rot occurrence. Thirty diseased 4-year-old GCG plants with typical above-ground wilting were collected: 15 from Tonghua County (41°15′ N, 126°25′ E) and 15 from Liuhe County (41°54′ N, 125°17′ E). Diseased GCG showed yellow-brown, withered, drooping leaves, and severe infections caused whole-plant death. Root surfaces bore scattered irregular rust-brown necrotic spots; mild infections displayed faint rust speckles, while severe lesions expanded into large rotten patches with rough, corky, easily detached epidermal tissue. The GCG fields feature high-organic-matter (≥3%) dark brown sandy loam soils (pH 5.5–6.5) with good drainage. Long-term monoculture leads to pathogen buildup and severe replanting disorders, causing far higher rust root rot incidence than forest-cultivated ginseng (LXSS). We also sampled 45 LXSS plants aged 4–8 years (15 plants per age group) from Baishan City (41°21′ N, 126°7′ E). LXSS grows in thick-humus dark brown forest loam (pH 5.5–6.5, organic matter ≥ 5%) with loose texture and excellent aeration, forming a stable rhizosphere environment that drastically limits rust root rot outbreaks.

### 2.2. Isolation of Fungi

Ginseng root lesions were cut into small pieces (1 cm × 1 cm), and each piece was placed in a Petri dish containing potato dextrose agar (PDA) medium supplemented with 100 ppm penicillin. After 7–10 days of culture in an incubator at 28 °C, aerial hyphae grew around the sample. A small amount of hyphal material was transferred to a new Petri dish of PDA medium and was cultured at 28 °C for a further 7–10 days [25]. This step was repeated 2–3 times to obtain two strains of fungi, SYM-1 and SYM-2.

### 2.3. Preparation of Fungal Spore Suspension

Sterile water (1 mL) was added to SYM-1 or SYM-2 colonies and the spores were gently scraped. Using a pipettor, water containing loose spores was transferred to a 1.5 mL sterile centrifuge tube and mixed thoroughly. The absorbance of the spore suspension at 600 nm was measured using a microplate reader (Thermo Fisher Scientific, Waltham, MA, USA). When OD_600_ = 0.5, the spore suspension was adjusted to 1 × 10^6^ spores·mL^−1^, and 1 μL of this suspension was added to 99 μL of sterile water to obtain a 100× diluted spore suspension. Each assay used a 50 μL aliquot of the working spore suspension, containing approximately 5 × 10^4^ spores. No bacteria were added in the experiment. The method was verified according to previous studies [26].

### 2.4. Preparation of Fungal Hyphae Liquid

PDA medium containing strain SYM-1 or SYM-2 was transferred into 150 mL of potato dextrose broth PDB liquid medium using a puncher and cultured at 28 °C and 180 rpm for 4–6 days. The culture liquid was then filtered with gauze into a 50 mL centrifuge tube, and the morphological characteristics were observed under a light microscope (Carl Zeiss, Oberkochen, Germany) (Figure S1). The absorbance of the culture liquid at 600 nm was measured by enzyme calibration. When the OD_600_ = 0.9, the liquid was diluted to 100× to prepare a fungal hyphae liquid.

### 2.5. Determination of Fungal Infectivity

Nine healthy 4-year-old ginseng were selected from a garden in Tonghua County. These healthy ginseng samples were washed with sterile water, and then the roots were cut with a knife to give a 3 cm wound, and the wound was plugged with cotton [27]. Each root received three wounds (inoculation points). The fungal suspension was filtered through gauze into a 50 mL centrifuge tube, and morphological features were observed under a light microscope (Figure S1). Three ginseng samples were then treated with 50 μL of SYM-1 and three with a SYM-2 spore suspension, which was injected into the cotton. The remaining three ginseng were used as the control group and were treated with sterile water. These ginseng samples were then planted in sterile soil and were cultured at 25 ± 3 °C with a 16 h light/8 h dark photoperiod and 70% relative humidity for 15–30 days. All determinations and assays were carried out with at least three independent biological replicates, and the infection of the lesions was observed.

### 2.6. Identification of Fungi Responsible

After the activation of strains of SYM-1 or SYM-2, appropriate amounts of mycelia were selected and placed into 1.5 mL centrifuge tubes. Liquid nitrogen was added and the mycelia were ground to a powder using a grinding rod. DNA (Deoxyribonucleic acid) was extracted from the fungi using a Sangon Biotech kit. The primers ITS1 (5′-TCCGTAGGTGAACCTGCGG-3′) and ITS4 (5′-TCCTCCGCTTATTGATATATGC-3′) were used for the amplification of the ITS region. After DNA amplification using PCR (Polymerase Chain Reaction), the products were separated by agarose gel electrophoresis for 30 min, and the results were observed by a gel imager. The PCR procedure and system are shown in Table S1. The non-impurity bright band samples were screened and sent to Shanghai Shenggong Biotechnology Co., Ltd. (Shanghai, China), for sequencing analysis. After the DNA sequence was obtained, an NCBI Blast search was conducted for other known species sequences, the sequences were aligned, and a phylogenetic tree was constructed in MEGA to identify the fungi [28].

### 2.7. Determination of Ginsenoside Concentration in Ginseng Rhizospheric Soil

The surface soil was excavated layer by layer from the base of the plant using a soil knife. After the ginseng root system was completely excavated, the surface soil was gently shaken off. The residual soil in the root was the rhizospheric soil, which was collected with a brush and placed in a sterile centrifuge tube. A mass of 1 g of ginseng rhizospheric soil was placed into a 10 mL centrifuge tube with 5 mL of chromatographic methanol, and ultrasonic extraction was performed for 40 min. The sample was then centrifuged at 8000 rpm for 10 min, and 5 mL of methanol was added to the remaining residue for ultrasonic extraction for 40 min. The two extraction supernatants were then combined and were concentrated using a rotary evaporator (IKA, Staufen, Germany). A volume of 5 mL of chromatographic methanol was added to the tube in the evaporated rotary evaporator to rinse the wall of the bottle. After rotary evaporation, the solvent was removed and chromatographic methanol was added. The solution was filtered through a 0.22 mm filter membrane and reduced to 1 mL. Quantitative analysis was then performed using the MRM mode of UPLC–MS/MS (Shimadzu, Kyoto, Japan). The liquid phase conditions were as follows: mobile phases A and B were acid water (0.1% formic acid) and acetonitrile, respectively. The temperature of the column box was maintained at 35 °C. The chromatographic column was C_18_ (Shim-pack GIST C_18_, 2 μm, 100 × 2.1 mm). The flow rate was 0.2 mL/min, and the injection volume was 10 μL. A gradient elution method was used, and the gradient elution conditions were 0–12 min, 5–95% B; 12–13 min, 95% B. MS analysis conditions were as follows: using negative ion mode, the compound was detected using MRM mode, interface temperature 300 °C, DL temperature 250 °C, heating block temperature 450 °C, drying gas flow rate 15 L·min^−1^, heating gas flow rate 5 L·min^−1^ [29]. Three replicates were set for each group of samples. The ginsenosides Rb_1_, Rd, Re, Rg_1_, and Rg_2_ were monitored using the Multiple Reaction Monitoring (MRM) mode. The specific MRM parameters for each ginsenoside are given in Table S2. The calibration curves of ginsenosides Rb_1_, Rd, Re, Rg_1_, and Rg_2_ are given in Table S3.

### 2.8. Growth Activity of Fungal Mycelia Following Treatment with Ginsenosides

A mass of 2 mg ginsenoside standard was dissolved in 20 μL of chromatographic methanol. To each well of a 96-well plate, 100 μL of fungal suspension, 99 μL of PDB liquid medium and 1 μL of ginsenoside standard were added. The initial concentration of ginsenoside (Rb_1_, Rd, Re, Rg_1_, and Rg_2_) was 512 μg·mL^−1^, and this was diluted into a concentration gradient of 6 concentrations. The control contained 100 μL of fungal suspension, 99 μL of PDB liquid medium and 1 μL of methanol. Each concentration in the gradient was repeated 3 times. The plates were sealed with sealing film and were cultured in an incubator at 28 °C for 72 h, and the absorbance of the culture at 600 nm was measured using a spectrophotometer every 12 h. Finally, the mycelium growth inhibition ratio (%) was calculated according to the formula [(Control OD − Treated OD)/Control OD] × 100% [26].

### 2.9. Germination of the Fungal Spore Suspension Following Treatment with Ginsenosides

All five ginsenoside standards (Rb_1_, Rd, Re, Rg_1_, Rg_2_, purity ≥ 98%) were purchased from Shanghai Yuanye Bio-Technology Co., Ltd. (Shanghai, China). A total of 2 mg of each individual ginsenoside standard was dissolved in 20 μL of chromatographic methanol. To each well of a 96-well plate, 100 μL of fungal spore suspension, 99 μL of sterile water and 1 μL of ginsenoside standard solution were added. The initial concentration of each ginsenoside standard solution was 512 μg·mL^−1^, which was serially diluted to generate six concentration gradients, with three biological replicates for each concentration. The control comprised 100 μL of spore suspension, 99 μL of sterile water and 1 μL of methanol. Plates were sealed with sealing film and were cultured in an incubator at 28 °C for 72 h, and the absorbance of the culture at 600 nm was measured using a spectrophotometer every 12 h. Finally, the growth promotion ratio (%) was calculated according to the formula [(Treated OD − Control OD)/Control OD] × 100% [28].

### 2.10. Statistical Analysis

All data were analyzed in SPSS 22.0 and graphics were created in GraphPad Prism 9.5. Normality of data distribution was tested using the Shapiro–Wilk test. If the data followed a normal distribution, an independent-samples *t*-test was used for comparison between two groups. For comparisons among three or more groups, one-way analysis of variance (ANOVA) was performed, followed by Tukey’s range test. Differences were considered statistically significant at *p* < 0.05 [30].

## 3. Results

### 3.1. Isolation, Identification, and Pathogenicity of GCG Fungi

**Figure 1:**
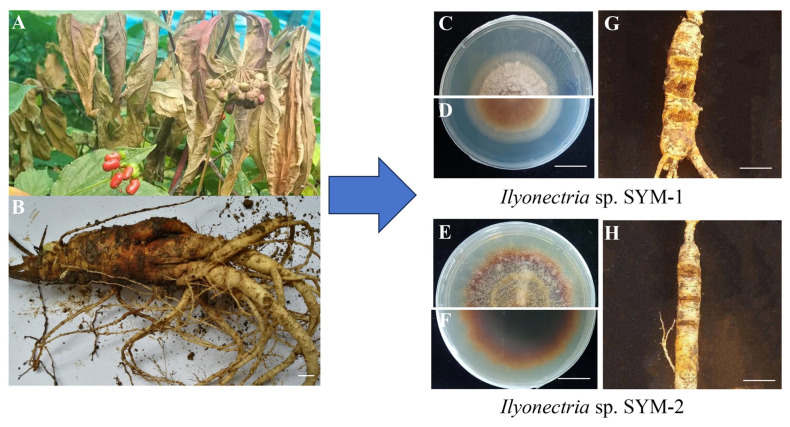
Morphology of ginseng infected with rusty root rot and colony morphology of isolated fungi. (**A**) The morphological characteristics of the aerial parts of ginseng infected with rusty root rot in the field. (**B**) The morphological characteristics of ginseng roots infected with rusty root rot in the field. (**C**) The morphological characteristics of SYM-1 mycelium. (**D**) The morphological characteristics of SYM-1 mycelium underside. (**E**) The morphological characteristics of SYM-2 mycelium. (**F**) The morphological characteristics of SYM-2 mycelium underside. (**G**) Morphology of ginseng root 30 days following infection with SYM-1. (**H**) Morphology of ginseng root 30 days following infection with SYM-2. Scale bar = 1 cm.

We conducted a field survey of 4-year-old GCG in Tonghua and Liuhe, and many of them were found to be infected with rusty root rot. We also surveyed 4–8-year-old LXSS in Baishan. We found that the incidence of rusty root rot in GCG was about 20–30%, while this disease was almost never found in 4–8-year-old LXSS. Samples of GCG plants infected with rusty root rot were collected (Figure 1A,B). Pathogens from the diseased ginseng roots were isolated using a streak culture method, and two fungi were obtained, named here SYM-1 (Figure 1C,D) and SYM-2 (Figure 1E,F). Morphological identification was performed for the two fungi isolates. On PDA medium, strain SYM-1 colonies were white with dense aerial mycelia, and the reverse side was pale to light yellow. Strain SYM-2 colonies were brown with abundant aerial hyphae, and the reverse side was dark brown. Conidia were hyaline, unicellular, ellipsoid to fusiform, and consistent with typical morphological features of the genus *Ilyonectria* spp. In order to assess the infectivity of SYM-1 and SYM-2 in ginseng, spore suspensions of SYM-1 and SYM-2 were used separately to infect healthy ginseng roots. After 30 dpi of culture at 28 °C, the infected roots all appeared significantly reddish to the naked eye, with the area infected with SYM-1 being larger than that of SYM-2. In addition, the root infected with SYM-1 had a deeper color than that infected with SYM-2. This suggests that SYM-1 has a higher infectivity in ginseng than does SYM-2 (Figure 1). Therefore, SYM-1 and SYM-2 are pathogenic fungi of ginseng rusty root rot.

### 3.2. Identification of the Fungi

**Figure 2:**
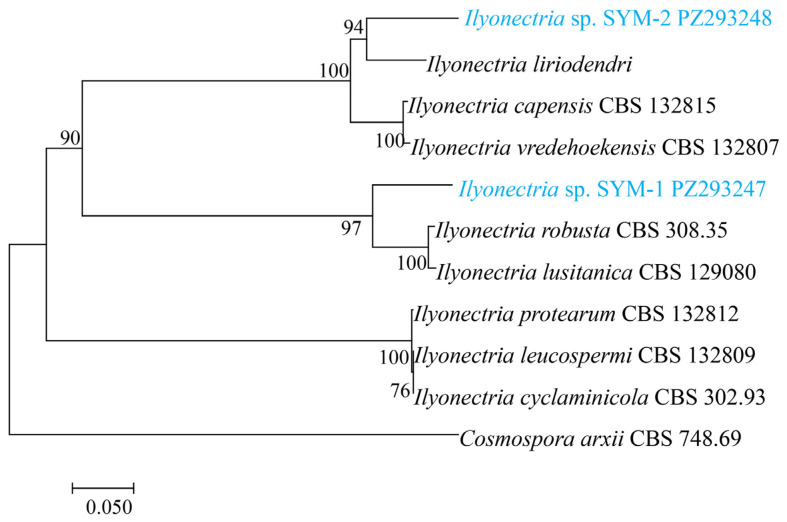
Phylogenetic tree reconstructed using DNA sequences from *Ilyonectria* sp. SYM-1 and *Ilyonectria* sp. SYM-2 and sequences downloaded from NCBI.

DNA was extracted from purified mycelia of SYM-1 and SYM-2, and its concentration was determined. The ITS region was amplified by PCR and sequenced. For phylogenetic analysis, only sequences from type strains or reliably identified strains of *Ilyonectria* retrieved from NCBI GenBank were included. Sequences were aligned using MAFFT, and a phylogenetic tree was constructed using the Maximum Likelihood (ML) method in MEGA 7 with the Kimura 2-parameter model; bootstrap values were calculated with 1000 replicates. ITS was used, as it is the universal fungal DNA barcode and is widely accepted for rapid genus- and species-level identification in *Ilyonectria*. Based on sequence similarity and phylogenetic analysis, SYM-1 were closely related to *Ilyonectria robusta and Ilyonectria lusitanica* but with only 97% bootstrap support. SYM-2 were closely related to *Ilyonectria liriodendri*, but with only 94% bootstrap support. Therefore, both isolates were identified at the genus level as *Ilyonectria* sp. SYM-1 and *Ilyonectria* sp. SYM-2 (Figure 2). All the purified pathogenic *Ilyonectria* spp. obtained in this study have been registered in GenBank, SYM-1 accession number is PZ293247, SYM-2 accession number is PZ293248.

### 3.3. Effect of Ginseng Rusty Root Rot Infection on Ginsenoside Concentration in Ginseng Rhizospheric Soil

**Figure 3:**
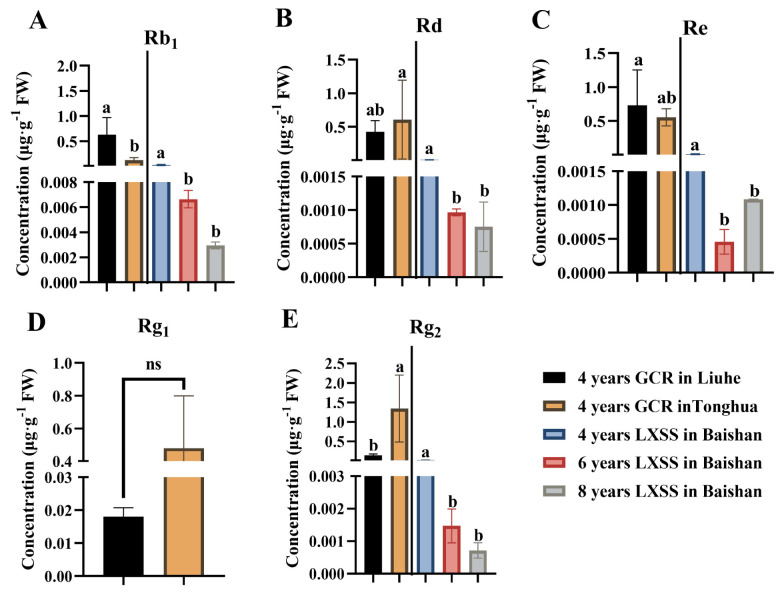
Quantitative analysis of ginsenosides in ginseng rhizospheric soil from different regions and growth years. (**A**) to (**E**) represent the concentrations of compounds Rb_1_, Rd, Re, Rg_1_, and Rg_2_, respectively. Soil samples were obtained from 4-year-old GCG (Liuhe County, Tonghua City) and 4-8-year-old LXSS (Baishan City, Jilin Province). Bars represent mean values, and error bars represent standard error. One-way ANOVA followed by Tukey’s HSD multiple comparison test was used for statistical analysis. Identical lowercase letters indicate no significant difference, whereas different letters indicate significant differences (*p *< 0.05).

UPLC–MS/MS was used to analyze ginsenosides in rhizospheric soil root exudates around GCG. The results showed that ginsenosides Rb_1_, Rd, Re, Rg_1_ and Rg_2_ and were all found in the GCG rhizospheric soil (Figure S2). Our results showed that the concentration of Rb_1_ in the rhizospheric soil of GCG collected in Liuhe was significantly higher than that of GCG planted in Tonghua, with concentrations of 628.99 ± 340.2 ng·g^−1^ FW and 125.48 ± 48.54 ng·g^−1^ FW, respectively (Figure 3A). However, the concentrations of the ginsenoside Rg_2_ were found to be higher in Tonghua (1341.46 ± 859.91 ng·g^−1^ FW) than in Liuhe (Figure 3E).

Next, UPLC–MS/MS was used to analyze the rhizospheric soil of 4–8-year-old LXSS from Baishan City. We found that the ginsenoside Rg_1_ was not detected at all in LXSS rhizospheric soil (Figure S3). The concentrations of ginsenoside in the rhizospheric soil of 4-year-old LXSS were the highest of all LXSS ages tested but decreased with increasing age of LXSS. The concentration of the ginsenoside Rb_1_ in the rhizospheric soil of LXSS was the highest of all the five ginsenosides across all ages of plants, with concentrations of 28.44 ± 11.75 ng·g^−1^ FW, 6.64 ± 0.70 ng·g^−1^ FW, and 2.94 ± 0.27 ng·g^−1^ FW, in 4-, 6- and 8-year-old plants, respectively (Figure 3A).

### 3.4. Inhibitory Effect of Ginsenosides on Mycelial Growth of Ilyonectria sp. SYM-1

**Figure 4:**
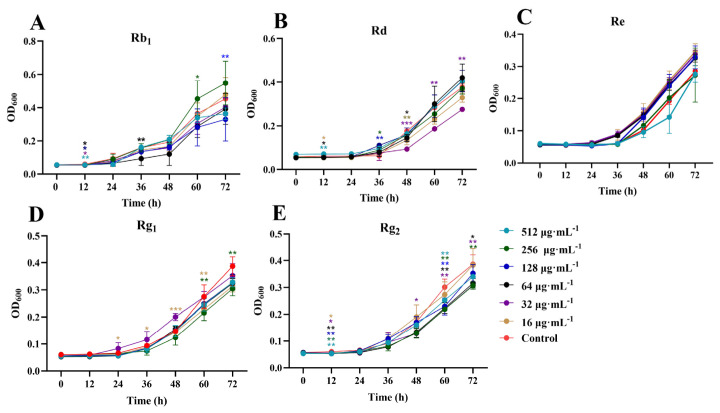
Effects of ginsenosides on the mycelial growth of *Ilyonectria* sp. The growth of SYM-1 mycelia in nutritive suspension over 72 h. The mycelia were treated independently with different concentrations of ginsenosides Rb_1_ (**A**), Rd (**B**), Re (**C**), Rg_1_ (**D**), or Rg_2_ (**E**). Independent-samples *t*-tests were used for comparison between the experimental and control groups, where *** denotes *p* < 0.001, ** denotes *p* < 0.01, and * denotes *p* < 0.05.

To determine whether ginsenosides have an inhibitory effect on the growth of *Ilyonectria* sp. SYM-1, a 96-well plate meter (OD_600_) was used to evaluate the concentration of hyphae in the nutrient fungal solution in the presence of different ginsenosides. The ginsenosides Rb_1_, Rd, Rg_1_, and Rg_2_ all had an inordinately inhibitory effect on mycelial growth. After co-culture with 128 μg·mL^−1^ Rb_1_ at 72 h, the growth of *Ilyonectria* sp. SYM-1 was potently inhibited (*p* < 0.01) (Figure 4A). Rd had a significantly inhibitory effect on the mycelial growth of *Ilyonectria* sp. SYM-1 at concentrations 32 μg·mL^−1^, with the inhibitory effect reaching a peak at 48 h (*p* < 0.001), with the highest inhibition rate of 45.98 ± 6.36% (Figure 4B). After treatment with 16 μg·mL^−1^ Rg_1_ for 48 h, the inhibition of *Ilyonectria* sp. SYM-1 was 47.99 ± 1.39% (Figure 4D). Rg_2_ at concentrations of 32 μg·mL^−1^ or 64 μg·mL^−1^ had a significant inhibitory effect at 72 h (*p* < 0.01), with inhibition rates of 24.94 ± 14.94% and 24.13 ± 4.63%, respectively (Figure 4E).

### 3.5. Promotional Effect of Ginsenosides on Spore Germination of Ilyonectria sp. SYM-1

**Figure 5:**
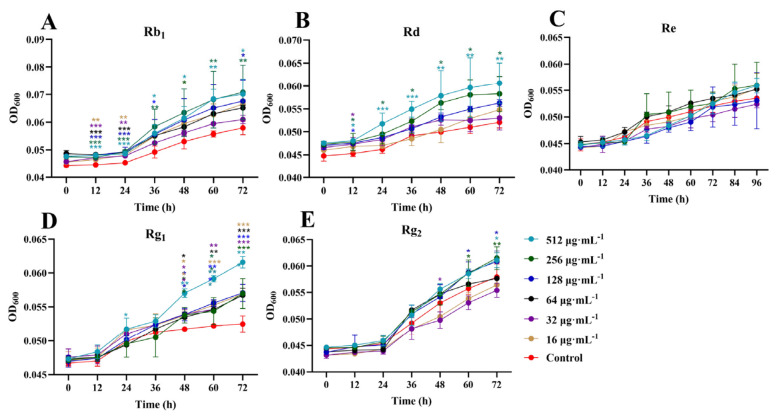
Effects of ginsenosides on spore germination of *Ilyonectria* sp. SYM-1 grown in non-nutritive suspension over 72 h. The spores were independently treated with different concentrations of ginsenosides Rb_1_ (**A**), Rd (**B**), Re (**C**), Rg_1_ (**D**), or Rg_2_ (**E**). Independent-samples *t*-tests were used for comparison between the experimental and control groups, where *** denotes *p* < 0.001, ** denotes *p* < 0.01, and * denotes *p* < 0.05.

In order to determine whether these ginsenosides have an effect on spore germination of *Ilyonectria* sp. SYM-1, the concentration of spores in non-nutritive spore suspensions in the presence of different ginsenosides was evaluated using a 96-well plate meter (OD_600_). Five concentrations of ginsenoside Rb_1_ were found to promote spore germination in *Ilyonectria* sp. SYM-1 at 12–24 h (*p* < 0.001), but the promotion of spore germination gradually decreased with increasing treatment time (Figure 5A). Treatment with Rd at 512 μg·mL^−1^ had a continuous promotional effect on spore germination. At 36 h, the spore germination promotion was the most significant (*p* < 0.001), with a promotion rate of 55.93 ± 22.09% (Figure 5B). With increased treatment time, the promotional activity of different concentrations of the ginsenoside Rg_1_ on the spore germination of *Ilyonectria* sp. SYM-1 also increased. At 512 μg·mL^−1^, the ginsenoside Rg_1_ showed continuous significant germination promotional activity between 48 h and 72 h (*p* < 0.001), with rates between 22.96 ± 2.01% and 43.03 ± 1.51% (Figure 5D). The ginsenoside Rg_2_ also promoted spore germination at concentrations of 32–512 μg·mL^−1^, but the effect was weak (Figure 5E).

### 3.6. Inhibitory Effect of Ginsenosides on Mycelial Growth of Ilyonectria sp. SYM-2

**Figure 6:**
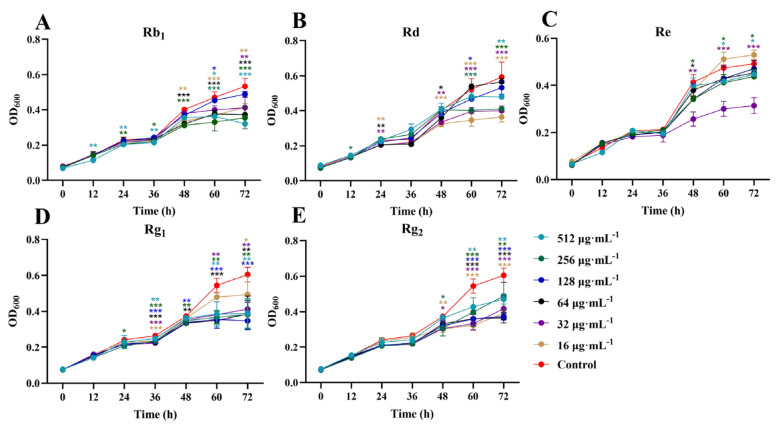
Effects of ginsenosides on mycelial growth of *Ilyonectria* sp. SYM-2 grown in nutritive suspension over 72 h. The mycelia were independently treated with different concentrations of ginsenosides Rb_1_ (**A**), Rd (**B**), Re (**C**), Rg_1_ (**D**), or Rg_2_ (**E**). Independent-samples *t*-tests were used for comparison between the experimental and control groups, where *** denotes *p* < 0.001, ** denotes *p* < 0.01, and * denotes *p* < 0.05.

Ginsenoside Rb_1_ showed significant inhibitory activity on the mycelial growth of *Ilyonectria* sp. SYM-2 at concentrations of 64 μg·mL^−1^ and 256 μg·mL^−1^ and from 48 to 72 h, and the inhibitory activity increased with time (Figure 6A). Of all the tested ginsenosides, Rd had the most significant effect on mycelial growth between 48 h and 72 h (*p* < 0.001) at 16 μg·mL^−1^, with inhibition rates between 19.17 ± 1.59% and 37.44 ± 1.41% (Figure 6B). A significant effect on the mycelial growth was seen following treatment with Re at 32 μg·mL^−1^ between 48 h and 72 h (*p* < 0.001), although the effect at other concentrations was weak (Figure 6C). Rg_1_ at a concentration of 128 μg·mL^−1^ had significant inhibitory activity between 36 and 72 h (Figure 6D). The effect of Rg_2_ on mycelial growth was strongest when the Rg_2_ concentration was between 16 μg·mL^−1^ and 128 μg·mL^−1^, and the reaction time was 60–72 h (Figure 6E).

### 3.7. Promotional Effect of Ginsenosides on Spore Germination of Ilyonectria sp. SYM-2

**Figure 7:**
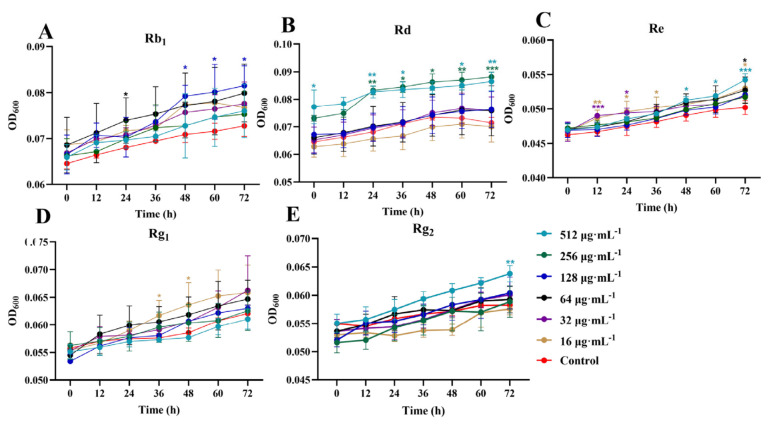
Effects of ginsenosides on spore germination of *Ilyonectria* sp. SYM-2 grown in non-nutritive suspension over 72 h. The spores were treated independently with different concentrations of ginsenosides Rb_1_ (**A**), Rd (**B**), Re (**C**), Rg_1_ (**D**), or Rg_2_ (**E**). Independent-samples *t*-tests were used for comparison between the experimental and control groups, where *** denotes *p* < 0.001, ** denotes *p* < 0.01, and * denotes *p* < 0.05.

When *Ilyonectria* sp. SYM-2 spores were grown with the ginsenosides Rb_1_ at 128 μg·mL^−1^, a significant promotion of spore germination was seen at 48–72 h (*p* < 0.05) (Figure 7A). Between 24 h and 72 h, the promotional activity of Rd at concentrations of 256 μg·mL^−1^ and 512 μg·mL^−1^ was significant, leading to a maximum spore germination promotion rate of 56.18 ± 3.91% (Figure 7B). Re at concentrations of 512 μg·mL^−1^, 32 μg·mL^−1^, or 16 μg·mL^−1^ following treatment for 12–60 h promoted spore germination significantly (Figure 7C). In addition, ginsenosides Rg_1_ and Rg_2_ showed no significant promoting effects on spore germination of *Ilyonectria* sp. SYM-2 (Figure 7D,E).

### 3.8. Pathogen Infection Reduced the Ginsenoside Concentration of GCG

**Figure 8:**
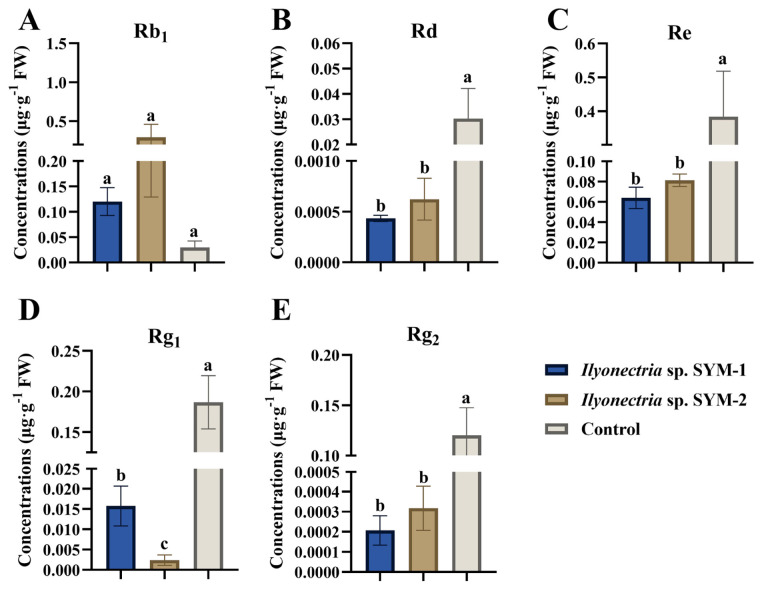
Quantitative analysis of the concentrations of ginsenosides in ginseng infected with *Ilyonectria* sp. SYM-1 or *Ilyonectria* sp. SYM-2. (**A**) to (**E**) represent the concentrations of compounds Rb_1_, Rd, Re, Rg_1_, and Rg_2_, respectively. The bar chart represents the average value, and the error line represents the standard error. The overall difference was assessed by one-way ANOVA, followed by Tukey’s HSD test for multiple comparisons. The same letter indicates that the difference is not significant, and different letters indicate that the difference is significant (*p* < 0.05).

The levels of ginsenosides Rb_1_, Rd, Re, Rg_1_ and Rg_2_ were evaluated in 4-year-old GCG infected with *Ilyonectria* sp. SYM-1 or *Ilyonectria* sp. SYM-2 (Figure S4). These results showed that the concentrations of Rd, Re, Rg_1_ and Rg_2_ in 4-year-old GCG inoculated with *Ilyonectria* sp. SYM-1 or *Ilyonectria* sp. SYM-2 were lower than the control group. Among them, after inoculation with *Ilyonectria* sp. SYM-1, the concentration of Rd was 0.43 ± 0.03 ng·g^−1^ FW; the concentration of Re was 63.98 ± 10.21 ng·g^−1^ FW, and the concentration of Rg_2_ was ng·g^−1^ FW, which, when compared with the control group, decreased by 97.61 ± 1.30%, 81.15 ± 10.06% and 99.81 ± 0.11%, respectively (Figure 8B–E).

In addition, after inoculation with *Ilyonectria* sp. SYM-2, the concentration of Rd was 0.62 ± 0.21 ng·g^−1^ FW, which was significantly decreased by 98.37 ± 0.79% compared with the control group (Figure 8B). The concentration of Re was 81.37 ± 6.19 ng·g^−1^ FW, which was significantly decreased by 77.23 ± 6.78% compared with the control group (Figure 8C). The concentration of Rg_2_ was 0.32 ± 0.11 ng·g^−1^ FW, which was significantly decreased by 99.72 ± 0.13% compared with the control group (Figure 8E). In addition, the concentration of Rg_1_ in GCG infected with *Ilyonectria* sp. SYM-1 was higher than that in GCG infected with *Ilyonectria* sp. SYM-2. The concentration of Rg_1_ in GCG infected with *Ilyonectria* sp. SYM-1 was 15.74 ± 4.92 ng·g^−1^ FW, and the concentration of Rg_1_ in ginseng infected with *Ilyonectria* sp. SYM-2 was 2.40 ± 1.28 ng·g^−1^ FW (Figure 8D).

## 4. Discussion

### 4.1. Ilyonectria spp. Fungi Are Causal Agents of Rusty Root Rot in Ginseng

Ginseng often suffers from soil-borne diseases that damage its root system, eventually leading to a significant reduction in yield and quality [31]. The prevalence of GRR is a major issue in ginseng cultivation [32]. Currently, studies have shown that *Cylindrocarpon* spp. and *Ilyonectria* spp. are the main pathogens causing ginseng rusty root rot [33,34]. In this study, two fungi, *Ilyonectria* sp. SYM-1 and *Ilyonectria* sp. SYM-2 belonging to the *Ilyonectria* spp., were isolated from the GCG tissue of rusty root rot. Through the infection experiment of healthy ginseng, we found that *Ilyonectria* sp. SYM-1 and *Ilyonectria* sp. SYM-2 were pathogenic fungi of ginseng rusty root rot, and the therapeutic ability of SYM-1 was higher than that of SYM-2. This is consistent with the previous research results showing that *Ilyonectria* spp. was a pathogenic fungus of ginseng rusty root rot [35]. However, this study only used the ITS region for molecular identification of pathogens. Due to the limited resolution of single gene markers in defining closely related species in the *Ilyonectria* species complex, these two isolates can only be identified to the genus level. In subsequent studies, we will use multi-site sequence markers such as translation elongation factor 1-α (*tef1-α*) and β-tubulin to achieve accurate species-level classification.

### 4.2. The Occurrence of Rusty Root Rot Was Related to the Ginsenoside Concentration in Secreted Root Exudates

Currently, a large number of studies have shown that there is a close relationship between plant root exudates and rhizospheric microorganisms [36,37]. Many soil-borne pathogens rely on host root exudates to regulate germination, attraction, and infection [38]. These exudates may attract or repel pathogenic fungi during their swimming phase but may also stimulate or inhibit the germination of non-swimming propagules [31]. Although ginseng root exudates are known to shape microbial communities and contribute to replanting problems, few studies have linked specific exudate components to *Ilyonectria* infection [39]. Here, we found that diseased GCG released significantly more ginsenosides into the rhizosphere than healthy 4–8-year-old LXSS. In addition, we found that with increasing cultivation years, the release of ginsenosides to the soil gradually decreases. Ginsenosides strongly promoted spore germination of *Ilyonectria* spp., suggesting they act as key chemoattractants and stimulants rather than simple defensive compounds. Notably, in vitro concentrations were over 1000-fold higher than rhizosphere soil levels to ensure clear phenotypic responses; field effects may be milder, but our core conclusion remains robust.

### 4.3. The Interaction Between Ginsenosides and Ilyonectria spp.

Plant pathogens cause severe losses in economically important crops by evolving diverse strategies to evade host defenses [40]. Meanwhile, plants rely on various defensive metabolites, especially secondary metabolites, to resist pathogen infection [41]. Ginsenosides are the main defensive compounds in ginseng and can mediate rhizosphere microbial interactions [42]. In addition, it has been suggested that specific concentrations of ginsenosides could be used as a medium for plant–fungal interaction, and in some cases, lower concentrations of ginsenosides had a higher antibacterial effect than did higher concentrations [43]. Previous studies mainly focused on crude ginsenoside mixtures, while the functions of individual monomers against *Ilyonectria* spp. remain unclear [44,45]. Our results showed that all tested individual ginsenosides exerted a consistent stage-specific dual effect on *Ilyonectria* sp. SYM-1 and SYM-2: they significantly inhibited mycelial growth in a dose-dependent manner but markedly promoted spore germination across the tested concentration range. This phenomenon was observed in both strains and most ginsenoside monomers, indicating it is a common characteristic of the ginseng–*Ilyonectria* interaction. Such complexity helps explain why ginseng is still vulnerable to rusty root rot despite containing abundant antimicrobial metabolites. Furthermore, ginsenosides are not just passive exudates but also participate in plant immune regulation and defense signaling; we speculate that *Ilyonectria* may secrete ginsenoside-transforming enzymes to metabolize ginsenosides, though the specific mechanism remains to be verified.

## 5. Conclusions

In this study, two pathogenic fungi *Ilyonectria* sp. SYM-1 and SYM-2 causing ginseng rusty root rot were isolated. Ginsenosides play dual roles: they suppress fungal mycelial growth but high levels of Rd and Rg_1_ promote pathogen spore germination and infection. Diseased garden-cultivated ginseng releases much more rhizospheric ginsenosides than healthy forest-cultivated ginseng, and fungal infection greatly reduces ginseng’s internal ginsenoside content. This study clarifies the chemical interaction between ginseng and *Ilyonectria* pathogens. Reducing rhizospheric ginsenoside secretion is a promising way to control rusty root rot, and these findings offer theoretical support for ginseng disease prevention and resistant germplasm breeding.

## Data Availability

The original contributions presented in this study are included in the article/Supplementary Materials. Further inquiries can be directed to the corresponding authors.

## Supplementary Materials

The following supporting information can be downloaded at: https://www.mdpi.com/article/10.3390/microorganisms14071484/s1, Figure S1: The morphological characteristics of mycelia of *Ilyonectria* sp. SYM-1 and *Ilyonectria* sp. SYM-2 were observed under a light microscope; Figure S2: UPLC–MS/MS spectra of ginsenosides Rb_1_, Rd, Rg_2_, Re, and Rg_1_ in the rhizospheric soil surrounding the roots of 4-year old GCR infected with rusty root rot; Figure S3: UPLC–MS/MS spectra of ginsenosides Rb_1_, Rg_1_, Rg_2_, Rd, and Re in rhizospheric soil surrounding the roots of 4-, 6-, and 8-year-old LXSS; Figure S4: UPLC–MS/MS spectra of ginsenosides Rb_1_, Rd, Re, Rg_1_, and Rg_2_ extracted from the rhizospheric soil surrounding the roots of 4-year-old GCR infected with either *Ilyonectria* sp. SYM-1 or *Ilyonectria* sp. SYM-2; Table S1: Fungal identification PCR procedure and system; Table S2: Specific MRM parameters for ginsenosides Rb_1_, Rd, Re, Rg_1_, and Rg_2_; Table S3: UPLC–MS/MS calibration curves for ginsenosides Rb_1_, Rd, Re, Rg_1_, and Rg_2_.

## Abbreviations

The following abbreviations are used in this manuscript:

GCG	Garden-cultivated ginseng
LXSS	Lin-Xia-Shan-Shen
GRR	Ginseng rusty root rot
DNA	DeoxyriboNucleic Acid
PCR	Polymerase Chain Reaction
PDA	Potato Dextrose Agar
PDB	Potato Dextrose Broth
MRM	Multiple Reaction Monitoring
ML	Maximum Likelihood
