# Supplementary material for: Ginsenosides in the Root Exudates of Ginseng Infected with Rusty Root Rot Improve the Infectivity of Pathogenic Ilyonectria Fungi
Source: Microorganisms. 2026 Jul 7;14(7):1484. doi: 10.3390/microorganisms14071484 (PMC13413680; doi:10.3390/microorganisms14071484)
Supplement: Supplementary file 1 [file microorganisms-14-01484-s001.zip › microorganisms-4393890-supplementary.pdf]

Supplementary material

Table S1 Fungal identification PCR procedure and system.

PCR system (25μL)		PCR program		
DNA	3.0 μL	94 ℃		5 min
dd H_2O	9.0 μL	94 ℃	30 x	15 s
Mix enzyme	15.0 μL	55 ℃		15 min
ItS1	1.5 μL	72 ℃		30 s
ItS4	1.5 μL	72 ℃		5 min

Table S2 Specific MRM parameters for ginsenosides Rb_1, Rd, Re, Rg_1, and Rg_2.

NO.	Ginsenoside	Polarity	Precursor ion	Production	Q1 Pre bias (V)	CE (V)	Q3 pre Bias (V)
1	Rb_1	1108.55	179.15	9.0	38.0	50.0	17.0
		1108.55	119.20	9.0	38.0	54.0	10.0
		1108.55	221.30	9.0	48.0	55.0	20.0
2	Rd	945.45	621.45	9.0	22.0	44.0	34.0
		945.45	783.50	9.0	22.0	40.0	20.0
		945.45	119.05	9.0	36.0	50.0	20.0
3	Re	945.45	119.1	9.0	22.0	46.0	10.0
		945.45	101.15	9.0	22.0	54.0	13.0
		945.45	161.25	9.0	24.0	45.0	15.0
4	Rg_1	799.40	637.50	9.0	20.0	27.0	22.0
		799.40	161.30	9.0	28.0	31.0	30.0
		799.40	113.15	9.0	30.0	45.0	23.0
5	Rg_2	783.40	475.40	9.0	22.0	40.0	16.0
		783.40	637.40	9.0	22.0	32.0	24.0
		783.40	101.05	9.0	28.0	29.0	29.0

Table S3 UPLC-MS/MS calibration curves for ginsenosides Rb_1, Rd, Re, Rg_1, and Rg_2.

NO.	Ginsenoside	Calibration curves	R^2
1	Rb_1	Y=525607X+16031.9	0.999906
2	Rd	Y=640127X+18452.2	0.999935
3	Re	Y=299554X+9348.39	0.999641
4	Rg_1	Y=254303X+7229.13	0.999947
5	Rg_2	Y=2.73157e+006X+35199.5	0.999903

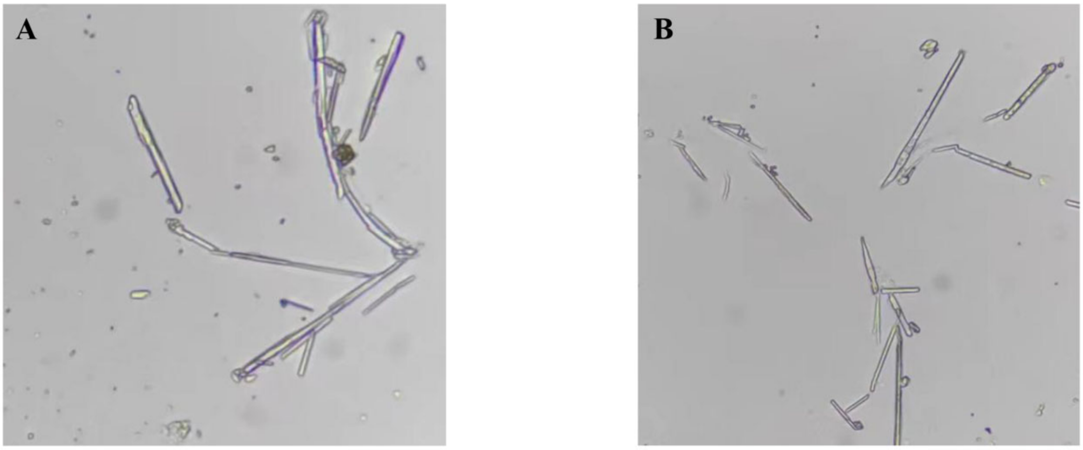

Figure S1 The morphological characteristics of mycelium of *Ilyonectria* sp. SYM-1 (A), and *Ilyonectria* sp. SYM-2 (B), were observed under a light microscope.

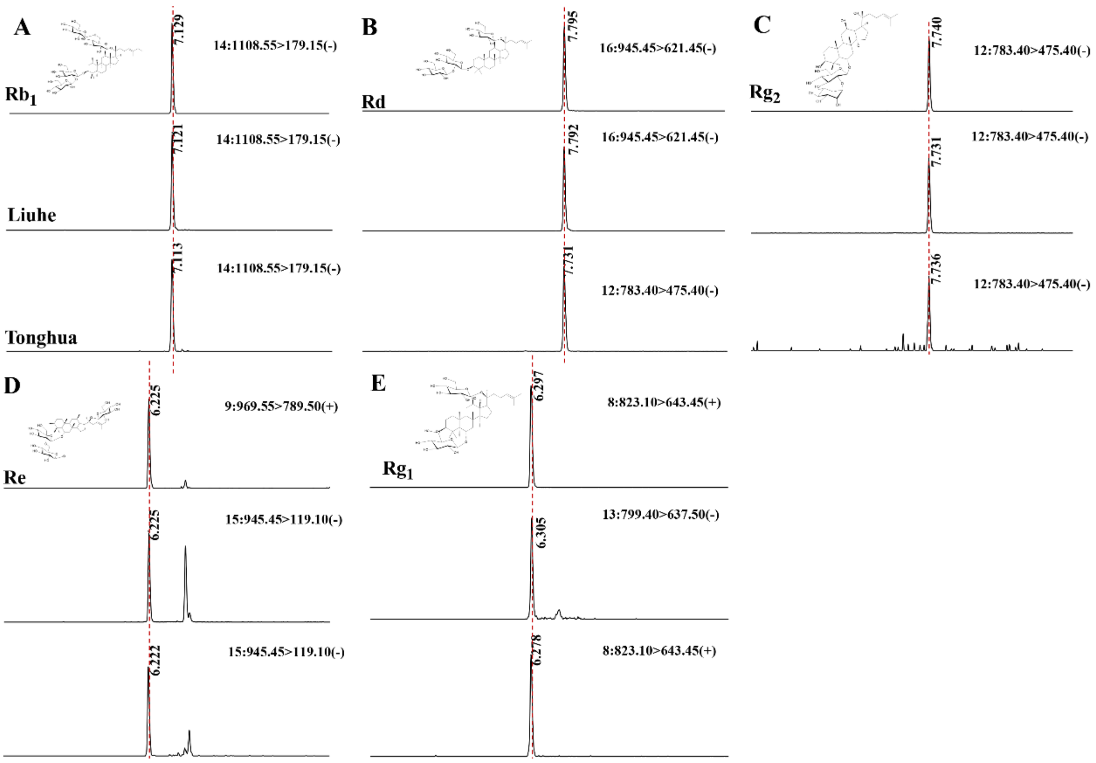

Figure S2 UPLC-MS/MS spectra of ginsenosides Rb_1 (A), Rd (B), Rg_2 (C), Re (D), and Rg_1 (E) in the rhizospheric soil surrounding the roots of 4-year old GCR infected with rusty root rot. Samples were taken from Liuhe County and Meihekou County, Jilin.

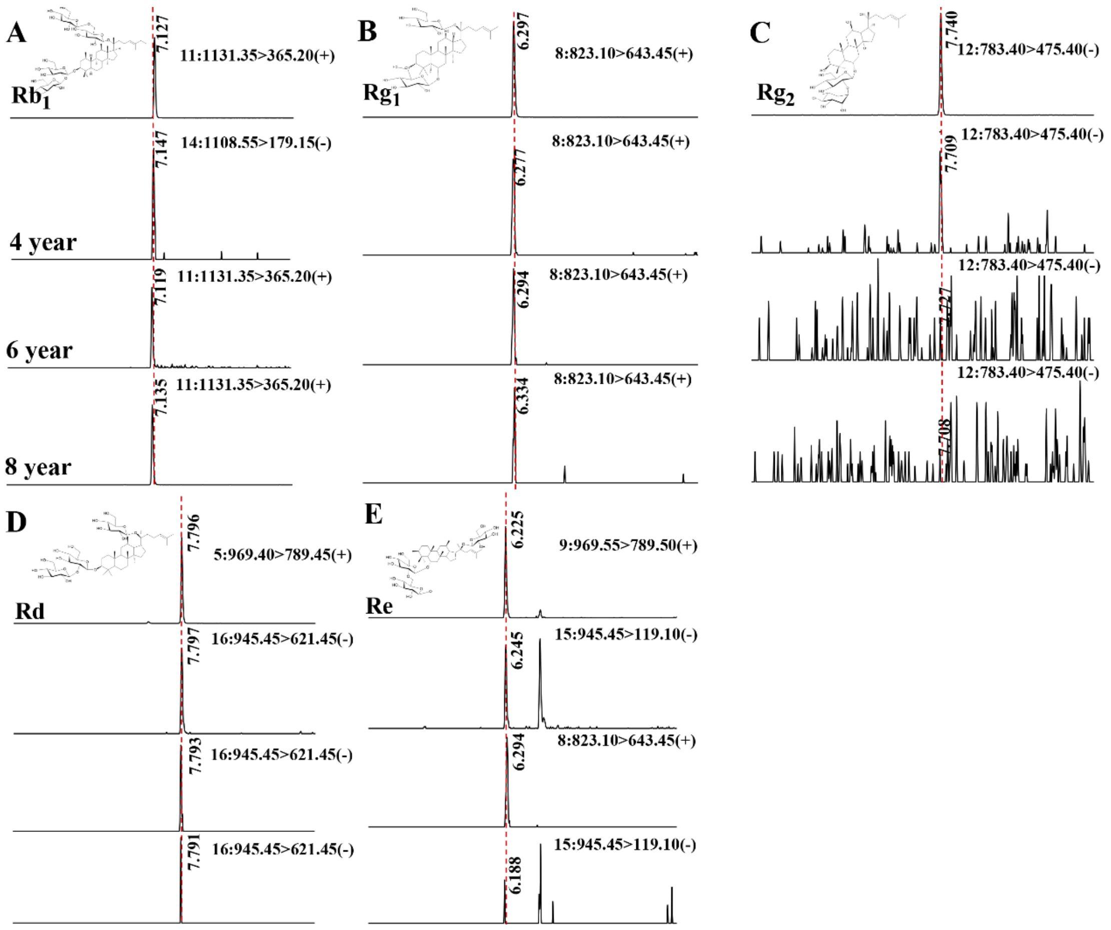

Figure S3 UPLC-MS/MS spectra of ginsenosides Rb_1 (A), Rg_1 (B), Rg_2 (C), Rd (D), and Re (E) in rhizospheric soil surrounding the roots of 4-, 6-, and 8-year old LXSS. Samples were taken in Baishan, Jilin.

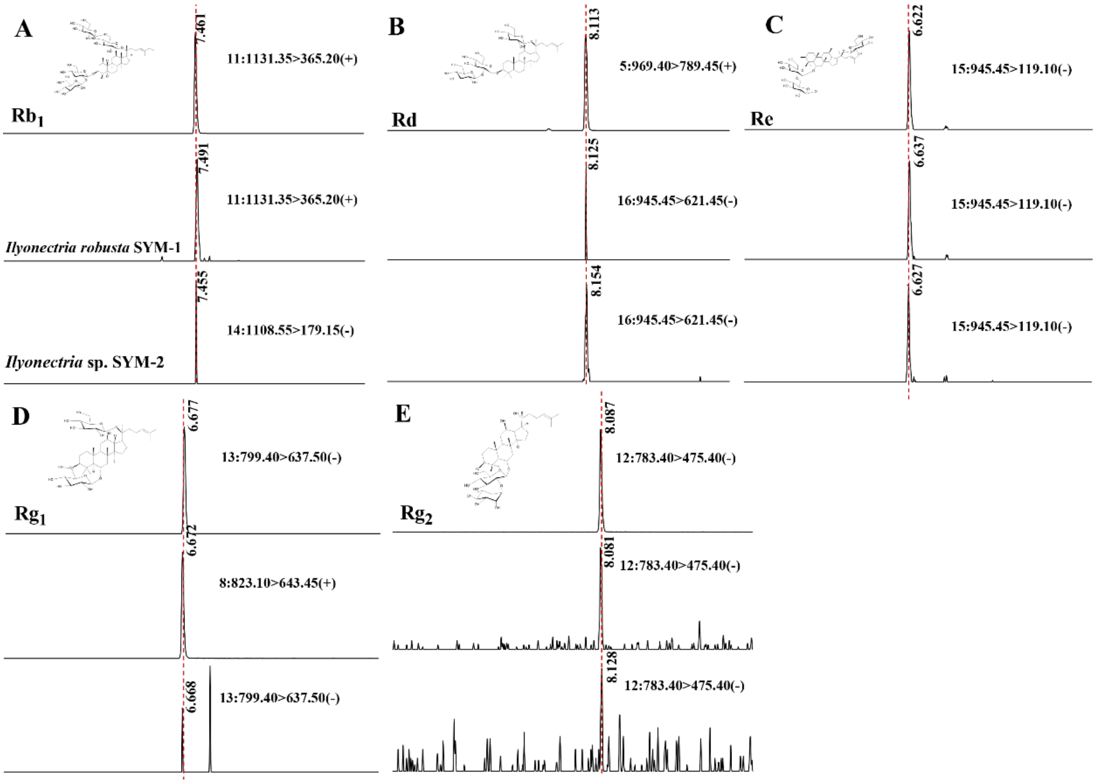

Figure S4 UPLC-MS/MS spectra of ginsenosides Rb_1 (A), Rd (B), Re (C), Rg_1 (D), and Rg_2 (E) extracted from the rhizospheric soil surrounding the roots of 4-year old GCR infected with either *Ilyonectria* sp. SYM-1 or *Ilyonectria* sp. SYM-2.

ITS sequencing sequence

>*Ilyonectria* sp. SYM-1

TCTCCGTTGGTGAACCACCAGCGGAGGGATCATTACCGAGTTTACAACTCCCAACCCC
TGTGACCATATTGTTGCCTCGGCGGTGCCCGTTTCGGCGGCCCGCCAGAGGACCCAAA
CCCTGTATTAAAGTATTCTTCTGAGTAAATGATTAAATCAATCAAAACTTTCAACAACGG
ATCTCTTGGCTCTGGCATCGATGAAGAACGCAGCGAAATGCGATAAGTAATGTGAATTG
CAGAATTCAGTGAATCATCGAATCTTTGAACGCACATTGCGCCCGCCAGTATTCTGGCG
GGCATGCCTGTCCGAGCGTCATTTCAACCCTCAAGCCCCCGGGCTTGGTGTTGGAGAC
CGGCAAGCCCTCCGGGGCACGCCGCCTCCCAAATTTAGTGGCGGTCTCGCTGTAGCTT
CCTCTGCGTAGTAGCACACCTCGCACTGGGAAACAGCGTGGCCACGCCGTAAAACCCC
CCACTTCTGAAAGGTTGACCTCGGATCAGGTAGGAATACCCGCTGAACTTAAGCATATC
AATAAGCGGAGGA

>*Ilyonectria* sp. SYM-2

TCCCCGTATGGTGACCTGCGGAGGGATCATTACCGAGTTTACAACTCCCAAACCCCCCC
CTGTGCGGCGGTGTCTGTTTCGGCAGCCCGCCAGAGGACCCAAACCCTAGATTACATT
AAAGCATTTTCTGAGTCAATGATTAAATCAATCAAAACTTTCAACAACGGATCTCTTGG
TTCTGGCATCGATGAAGAACGCAGCGAAATGCGATAAGTAATGTGAATTGCAGAATTC
AGTGAATCATCGAATCTTTGAACGCACATTGCGCCCGCCAGTATTCTGGCGGGCATGCC
TGTCCGAGCGTCATTTCAACCCTCAAGCCCCCGGGCTTGGTGTTGGAGATCGGCGAGC
CCCCCGGGGCGCGCCGTCTCCCAAATATAGTGGCGGTCCCGCTGTAGCTTCCTCTGCGT
AGTAGCACACCTCGCACTGGGAAACAGCGTGGCCACGCCGTAAAACCCCCCACTTCT
GAAAGGTTGACCTCGGATCAGGTAGGAATACCCGCTGAACTTAAGCATATCAATAAGC
GGAGGA
